# Impaired intratumoral natural killer cell function in head and neck carcinoma

**DOI:** 10.3389/fimmu.2022.997806

**Published:** 2022-10-20

**Authors:** Dalila Mele, Greta Pessino, Giuseppe Trisolini, Alberto Luchena, Marco Benazzo, Patrizia Morbini, Stefania Mantovani, Barbara Oliviero, Mario U. Mondelli, Stefania Varchetta

**Affiliations:** ^1^ Division of Clinical Immunology and Infectious Diseases, Fondazione Istituto di Ricovero e Cura Carattere Scientifico (IRCCS) Policlinico San Matteo, Pavia, Italy; ^2^ Unit of Immunology and General Pathology, Department of Molecular Medicine, University of Pavia, Pavia, Italy; ^3^ Division of Otorhinolaryngology, Department of Surgery, Fondazione Istituto di Ricovero e Cura Carattere Scientifico (IRCCS) Policlinico San Matteo, Pavia, Italy; ^4^ Department of Clinical, Surgical, Diagnostic and Pediatric Sciences, University of Pavia, Pavia, Italy; ^5^ Pathology Unit, Department of Molecular Medicine, University of Pavia, Pavia, Italy; ^6^ Department of Pathology, Fondazione Istituto di Ricovero e Cura Carattere Scientifico (IRCCS) Policlinico San Matteo, Pavia, Italy; ^7^ Department of Internal Medicine and Therapeutics, University of Pavia, Pavia, Italy

**Keywords:** tumor microenvironment, HNSCC, natural killer cells, GITR, PD-1, NKG2C, adaptive NK cells

## Abstract

Natural killer (NK) cells are emerging as unique players in the immune response against cancer; however, only limited data are available on tumor infiltrating NK cells in head and neck squamous cell carcinoma (HNSCC), one of the most common cancer. Occurrence of HNSCC is closely related to the immune microenvironment, and immunotherapy is increasingly being applied to this setting. However, the limited success of this type of treatment in this tumor calls for further investigation in the field.

Surgical HNSSC specimens of 32 consecutive patients were mechanically and enzymatically dissociated. Tumor cells were separated from infiltrating cells by short centrifugation and infiltrating NK cells were phenotypically and functionally characterized by multiple antibody staining and flow cytometry. Tumor infiltrating NK cells in HNSCC showed a peculiar phenotype predominantly characterized by increased NKG2A and reduced Siglec-7, NKG2D, NKp30 and CD16 expression. This phenotype was associated with a decreased ability to perform antibody-dependent cellular cytotoxicity (ADCC). However, NK, CD4 and CD8 shared an increment of glucocorticoid-induced tumor necrosis factor-related (GITR) costimulatory receptor which could be exploited for immunotherapy with agonistic anti-GITR antibodies combined with checkpoint inhibitors.

## Introduction

Head and neck squamous cell carcinoma (HNSCC) is one of the most common cancer with 878348 new cases and 444347 deaths in 2020 worldwide (1). HNSCC includes carcinoma originating from oral cavity, nasopharynx, hypopharynx, oropharynx and larynx, is closely associated to alcohol abuse and tobacco smoke and it may be associated with infection with oncogenic strains of human papillomavirus (HPV), such as HPV-16 and HPV-18. HPV positive HNSCC have a better prognosis than HPV-negative (2, 3). Treatment depends on the stage and the site of the disease, primary treatment options are surgical resection followed by chemotherapy and radiotherapy. The monoclonal antibody Cetuximab directed against epidermal growth factor receptor (EGFR) is used alone or in combination with chemotherapy, for the treatment of patients with recurrent or metastatic disease (4). Recently, the immune checkpoint inhibitors Nivolumab and Pembrolizumab have also been approved for the treatment of cisplatin-refractory recurrent or metastatic HNSCC (5, 6). Most patients have an advanced stage disease at diagnosis, because of the lack of important symptoms or reduced symptom awareness (7) and a local recurrence and lymphnode metastasis are frequently observed.

Unfortunately, five year survival rate is quite low, (56%-62%) and it has modestly improved over the years (8–10). A better survival is associated with the presence of tumor immune infiltrate (11–13), as shown by patients with a tumor microenvironment enriched in tumor infiltrating lymphocytes (TIL). Interestingly, most HPV positive HNSCC tumors are highly infiltrated by lymphocytes and have the best survival rates (13, 14), suggesting that the number of TIL plays an important role in the control of tumor growth. A recent study has shown that tumor immune microenvironment (TIME) in HNSCC is associated with immune checkpoint therapy response and better prognosis (15). Furthermore, the presence of defective NK genes related with NK cell cytotoxicity pathway and phenotypes, were associated with higher risk of developing cancer (15). Increasing data have shown that NK cells are not merely innate effector cells, but they are fundamental in the recruitment and activation of adaptive immunity, through the secretion of IFNγ which induces activation of T helper cells (15, 16). Moreover, in recent years different studies supported an increasing potential of NK cells in the control of different types of cancers, including HNSCC, where a favorable prognosis has been associated with NK cell infiltration (17–21).

However, a number of immune escape mechanisms are exploited by tumors to avoid NK cell recognition, thus a better knowledge of peripheral and intratumoral NK cell profile in patients with HNSCC is needed to identify possible therapeutic targets.

The activity of NK cells is finely tuned by several membrane receptors able to switch the NK cell function towards activation or inhibition after ligand recognition and interaction (22). The low affinity FcγRIIIA (CD16) is one of the most potent activating receptors of NK cells that mediates antibody-dependent cell-mediated cytotoxicity (ADCC) (23). It is associated with ITAM-containing

CD3 ζ-chain and its activation allows NK cells to recognize and kill antibody-coated target cells *via* ADCC and to release immunoregulatory cytokines.

Spontaneous NK cell lytic activity is mediated by several activating receptor-ligand axes, including the natural killer group 2, member D (NKG2D) activating receptor, and its ligands. NKG2D is a dimeric, type II membrane protein constitutively expressed by NK cells in humans, as well as by almost all CD8+ αβ T cells and γδ T cells (24). NKG2D receptors recognize several molecules, such as MHC class I chain-related molecules (MIC)A/B and the cytomegalovirus UL-16 protein (ULBP1-6), the expression of which is induced on the cell surface as a result of cellular stress conditions (25). Therefore, NKG2D plays a pivotal role in immune surveillance and antitumor immune responses (26–28). NKp30 is a natural cytotoxicity receptor constitutively expressed by NK cells whose engagement by its ligand transduces a strong activation signal to the cell (29). Siglec-7 is an inhibitory NK receptor whose reduced frequency is associated with decreased cell function in physiologic (30) and pathologic conditions (31–34).

PD-1 and NKG2A are checkpoint molecules that have been shown to harness NK and T cell immune responses in cancer (35). NKG2A forms a heterodimer with CD94. The NKG2A/CD94 complex binds to the non-classical MHC I molecule HLA-E in humans and transduces inhibitory signals which suppress NK and CD8+ T cell activity (36). Interestingly, NK cells are endowed by innate and adaptive properties, being able of long-term ‘memory-like’ immune responses (37, 38). Individual repertoire of NK cells may be shaped by environmental exposure, such as CMV infection, which is able to lead to a stable expansion of adaptive NK cell population characterized by the expression of NKG2C activating receptor (39–44). Sequence variations in CMV UL40-encoded peptides control the activation and expansion of adaptive NK cells (40–42), and the peptide variant VMAPRTLFL induces increased proliferation of NKG2C population compared with other CMV peptides (40, 41). Such expanded NK cells are identified by the lack of FcϵR1γ adaptor protein and display an altered receptor expression pattern characterized by reduced expression of NKp46, NKp30, CD16, Siglec-7 and NKG2A compared to conventional, FcεR1γ positive NK cells. Importantly, they have increased ability to perform ADCC and to produce IFN gamma (44–48).

In this study we explore immune infiltrating NK cell phenotype and function in intratumor, peritumor and peripheral blood of HNSCC patients and controls and evaluate NK cell function after CMV peptide-driven expansion of adaptive NK cells.

## Materials and methods

### Patients

Thirty-two individuals undergoing curative resection of HNSCC at the Fondazione IRCCS Policlinico San Matteo, Pavia, Italy were enrolled in the study. For each patient peripheral blood, tumor tissue, and, whenever possible, surrounding non-tumor tissue, were collected. Peripheral blood mononuclear cells collected from 19 healthy volunteers were used as control. **Table 1** shows the demographic and clinical features of patients and controls. The study protocol conformed to the ethical guidelines of the 1975 Declaration of Helsinki and was approved by the Institutional Review Board and Ethical Committee of Fondazione IRCCS Policlinico San Matteo, document number 20190063538.

**Table 1:** Clinicopathological characteristics of HNSCC patients and controls.

	HNSCC Patients	Control Group
**n (female/male)**	7/25	6/12
**Age range (y)**	(49-85)	(24-71)
**T- Primary tumor (n)** *T1* *T2* *T3* *T4*	3 6 14 9	
**N- Regional lymph node (n)** *N0* *N1* *N2a* *N3a* *N3b*	19 7 1 1 4	
**M- Distant metastasis (n)** *M0*	32	
**Stage grouping (n)** *I* *II* *III* *IVA*	2 3 16 11	
**Grade (n)** *G1* *G2* *G3*	1 29 2	
**Location (n)** Oral cavity Oropharynx Hypopharynx Larynx	17 2 4 9	

### Isolation of peripheral blood mononuclear cells and tissue-infiltrating lymphocytes

Peripheral blood mononuclear cell (PBMC) isolation was performed with Lympholyte (Cedarlane) density gradient (d=1.0770) centrifugation following the manufacturer’s instructions. To isolate tumor- (TIL) and non-tumor infiltrating lymphocytes (NIL) freshly dissected tissues were enzymatically and mechanically dissociated with the Tumor Dissociation Kit and gentle MACS Dissociator (Miltenyi Biotec), according to the manufacturer’s instructions. The resulting cell suspension was then filtered over a 70 µm cell strainer (Miltenyi Biotec) and centrifuged at 50xg for 1 min. The supernatant containing lymphocytes was washed, counted and resuspended in complete RPMI-1640 medium supplemented with 10% fetal bovine serum (FBS), 2mM L-glutamine and antibiotic antimycotic solution (Sigma-Aldrich) (RPMI complete). Cells were immediately used for immunophenotype staining or stimulated for functional analysis. Left-over PBMC, TIL and NIL were frozen in 90% FBS and 10% dimethylsulphoxide and stored in liquid nitrogen.

### Tumor cell lines

The K562 erythroleukemic and SW-480 colorectal carcinoma cell lines (ATCC) were grown in RPMI complete. The UM*-*SCC-22A squamous carcinoma cell line (Sigma-Aldrich) was maintained in complete Dulbecco’s Modified Eagle Medium (DMEM, ThermoFisher Scientific) supplemented with 10% FCS, 1 mM Sodium Pyruvate (Sigma-Aldrich), 1% antibiotic antimycotic solution, 1% L-glutamine and 1X non-essential amino acid (Sigma-Aldrich) (DMEM complete).

To establish *in vitro* primary HNSCC cell cultures, the cell pellet obtained after dissociation of resected tumor tissue was resuspended in CellGro SCGM (Cell Genix, Freiburg, Germany) supplemented with 10% fetal bovine serum (FBS; HyClone, GE Healthcare, South Logan, Utah, USA) and 1% antibiotic antimycotic solution (CellGro complete) and plated in 6-well plate. Viable tumor cells attached to the flask within 24 hours. Cultures at 75% to 100% confluence were selected for subculture by trypsinization with 0.05% trypsin/0.53 mM EDTA solution (Corning). The neoplastic origin of cultured cells was confirmed by an experienced histopathologist. Briefly, 50,000 formalin-fixed cells were stained with hematoxylin*-*eosin to evaluate in detail their morphologic features.

### Phenotypic analysis

2x10^6^ freshly isolated PBMC, TIL and, when available, NIL were used for flow-cytometric immunophenotypic analysis using a 12-color FACSCelesta (BD Biosciences). LIVE/DEAD^®^ Fixable Near-IR Dead Cell Stain Kit (Thermo Fisher Scientific) was used to determine cell viability. The immunophenotyping panel used to characterize the main lymphocyte subsets is given in **Supplementary Table 1**. Staining of nuclear trascription factor FcϵRIγ was performed using the Foxp3/Transcription Factor Staining Buffer Set (eBioscience) according to the manufacturer’s instructions.

### Cytotoxicity assay

Freshly isolated PBMC and TIL were stimulated overnight with IL12 (10 ng/ml), IL-15 (1 ng/ml) (Peprotech) and IL-18 (50 ng/ml, R&D System) in complete RPMI medium. After incubation, cells were harvested and co-cultured with K562 target cells at a 5:1 effector:target cell ratio for 5h at 37°C in the presence of anti-CD107a BV786 and BD GolgiPlug™ Protein Transport Inhibitor (BD). To assess the cytokine production, cells were fixed with BD Cytofix/Cytoperm (BD) and permeabilized with the BD Perm/Wash buffer (BD) and subsequently stained with anti-IFNγ PE (BD) and analyzed by FACSCelesta.

### Antibody-dependent cell-mediated cytotoxicity assay

Antibody-dependent cell-mediated cytotoxicity (ADCC) was performed using SW480 colon cancer cells as targets in the presence or absence of 10 μg/ml Cetuximab. Briefly, IL-12/15/18 pre-activated PBMC were incubated for 5 h with target cells at an E:T ratio of 5:1 in the presence of brefeldin A (GolgiPlug, BD). IFNγ production in NK cells was detected by intracellular staining and analyzed by FACSCelesta.

### *In vitro* expansion of PB-NK cells

Cryopreserved PBMC from 5 CMV+ HNSCC patients and 6 CMV+ control subjects were thawed, washed and rested for 2h in complete RPMI medium at 37°C. Subsequently, PBMC were seeded in 48 -well plates (10^6^ cells/well) and cultured for 12 days in complete medium supplemented with IL-2 250 IU/ml in the presence or absence of 400 μM synthetic peptides (HCMV-VMAPRTLFL or –VMAPRTLLL, peptides&elephants). IL-2-containing medium (250 IU/ml) was half-replaced at day 3, 6 and 9. At day 12, expanded NK cells were harvested and co-cultured with SW480 or UM-SCC-22A and/or autologous primary tumor cells at a ratio of 5:1 to assess their ADCC function. One ng/ml of IL-15 was added to culture on the day before functional assays

### HCMV serology

HCMV IgG serology was determined with DRG IgG CMV ELISA kit (DRG International).

### Statistical analysis

Statistical analysis was performed using the GraphPad Prism 8.4.3 software (GraphPad, La Jolla, CA, USA). Data distribution was examined by the D’Agostino & Pearson normality test. The Mann-Whitney U test was used to compare control subjects and HNSCC patients, more data sets were compared by the non-parametric Kruskal-Wallis followed by Dunn’s multiple test. The non-parametric Wilcoxon signed-rank test or the one-way Friedman followed by Dunn’s multiple comparison tests were used, as appropriate, to compare data within the same group. Correlations between variables were analyzed by Pearson’s rank correlation coefficient. Differences were deemed statistically significant when *p* ≤ 0.05.

## Results

### Circulating and tumor-infiltrating NK cells in HNSCC

**Figure 1:**
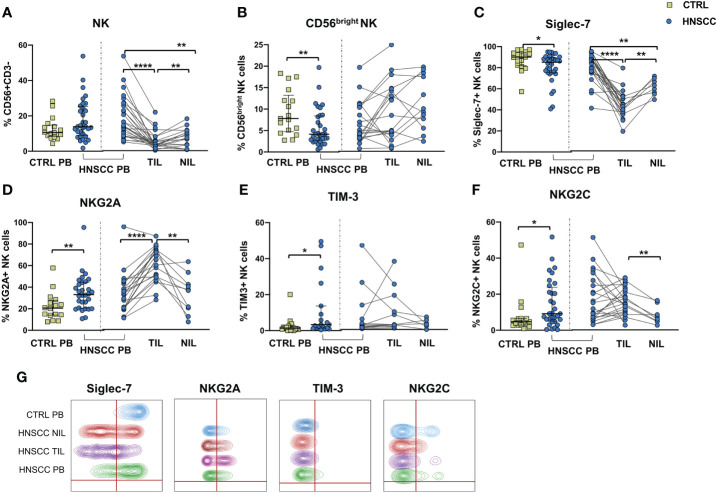
**(A)** Frequency of CD3^-^CD56 ^+^ NK cells and **(B)** CD56^bright^ NK cells in PBMC from control subjects (CTRL PB), and from HNSCC patients (HNSCC-PB) as matched tumor-infiltrating (TIL) and non- tumor-infiltrating lymphocytes (NIL). Proportion of **(C)** Siglec-7-, **(D)** NKG2A-, **(E)** TIM-3- and **(F)** NKG2C- positive NK cells. **(G)** Representative concatenated flow cytometry plots showing the proportion of NK cells expressing the indicated phenotypic markers in 6 representative CTRL PB, 6 HNSCC-PB, 6 HNSCC-TIL and 6 HNSCC-NIL. The Mann–Whitney U test was used to compare CTRL and HNSCC subjects. Paired data were analyzed by the Wilcoxon signed rank test. All plots include observations from 25th to 75th percentile. The horizontal line represents the median value. *p < 0.05, **p < 0.01, ****p < 0.0001.

We explored the quality of NK cell phenotype and function in peripheral blood, tumor and non tumor infiltrating lymphocytes (PB-NK, TIL-NK and NIL-NK respectively) in HNSCC tumor specimens. Tumor and non tumor tissues were scarcely infiltrated by NK cells, whereas T cells were largely predominant. Unfortunately, the number of cells retrieved from tissue was occasionally insufficient to allow performance of all the experiments planned. Specifically, the surrounding non-tumor tissue was often very scarcely infiltrated by immune cells. To allow clear data reporting, we showed paired data comparing PB-, TIL- and NIL- NK cell phenotype of HNSCC patients. Comparison between HNSCC patients and controls, showed that the proportion of circulating NK cells was similar, whereas these cells were strongly reduced in the non tumor and, particularly, in the tumor compartment compared with paired PB-NK (**Figure 1A**). The peripheral NK cell compartment was characterized by a reduced proportion of CD56^bright^ and Siglec-7-expressing NK cells in HNSCC subjects (**Figures 1B, C**). While no differences were observed in the CD56^bright^ subset distribution in tissues, the frequency of Siglec-7 positive NK cells was significantly reduced in TIL compared with paired PB and NIL (**Figure 1C, G**).

The frequency of circulating NK cells expressing NKG2A and TIM-3 was increased compared with controls (**Figures 1D, E, G**). NKG2A positive NK cells were further augmented inside the tumor tissue compared with PB and NIL NK cells (**Figure 1D**). The proportion of NK cells expressing NKG2C receptor was also increased in the peripheral blood of patients compared with control PB and in tumor tissue when compared with the non tumor compartment (**Figure 1F, G**).

**Figure 2:**
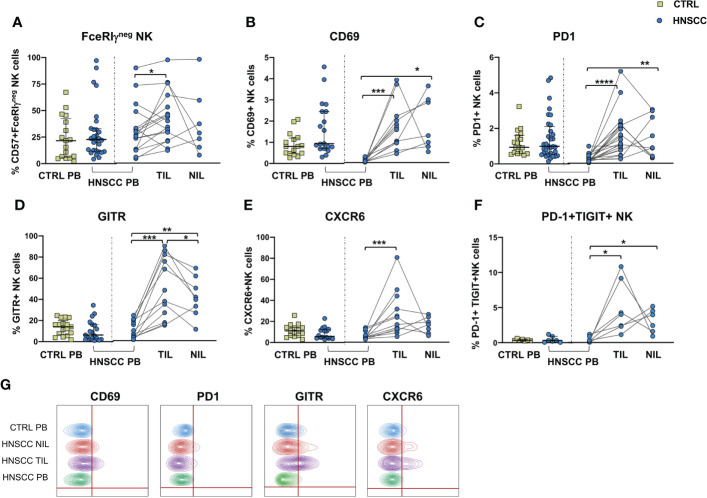
**(A)** Frequency of FcϵRIγ negative CD56^+^/CD57^+^ adaptive NK cells in PBMC from control subjects (CTRL PB), and from HNSCC patients (HNSCC-PB) as well as matched tumor-infiltrating lymphocytes (TIL) and surrounding non-tumor-infiltrating lymphocytes (NIL). Percentage of NK cells expressing **(B)** CD69, **(C)** PD-1 **(D)** GITR and **(E)** CXCR6. **(F)** Frequency of PD-1+TIGIT+ NK cells. **(G)** Representative concatenated flow cytometry plots showing the percentage of expression of the indicated phenotypic markers on CD3^-^CD56^+^ NK cells. The Mann–Whitney U test was used to compare CTRL and HNSCC subjects. Paired data were analyzed by the Wilcoxon signed rank test. *p < 0.05, **p < 0.01, ***p < 0.001, ****p < 0.0001.

**Figure 3:**
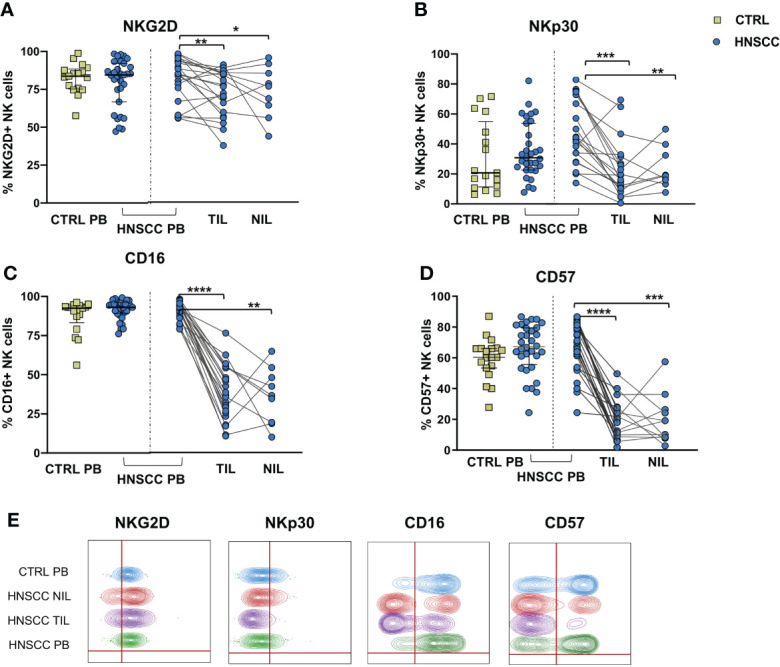
Frequences of NK cells expressing **(A)** NKG2D, **(B)** NKp30, **(C)** CD16 and **(D)** CD57 were determined in PBMC from control subjects (CTRL PB), and from HNSCC patients (HNSCC-PB) as well as matched tumor- (TIL) and surrounding non-tumor infiltrating lymphocytes (NIL). **(E)** Representative concatenated flow cytometry plots showing the percentage of expression of the indicated phenotypic markers on CD3^-^CD56^+^ NK cells. The Mann–Whitney U test was used to compare CTRL and HNSCC subjects. Paired data were analyzed by the Wilcoxon signed rank test. *p < 0.05, **p < 0.01, ***p < 0.001, ****p < 0.0001.

No differences in the frequency of circulating FcεRIγ negative adaptive/memory NK cell population were found in the two groups, despite there being a small, though statistically significant, increase in the tumor compartment compared with the periphery in HNSCC patients (**Figure 2A**). Other immune regulatory receptors were not differentially expressed in peripheral blood NK cells from patients and controls, but some of these molecules (CD69, PD-1, GITR and CXCR6) were significantly upregulated (**Figures 2B–E, G**) and other significantly reduced (NKG2D, NKP30, CD16 and CD57) (**Figures 3A–E**) in TIL-NK compared with circulating NK cells. Interestingly, the frequency of NK cells coexpressing PD-1 and TIGIT exhaustion markers was significantly increased in tumor compared with peripheral blood compartment (**Figure 2F**). TIGIT+ and NKp46+ NK cell frequencies did not differ between the compartments analyzed (**Supplementary Figure 1**). Thus, there were increased frequencies of NK cells coexpressing PD-1 and TIGIT in the tumor microenvironment. Significantly lower frequencies of NK cells expressing NKG2D, NKp30 and CD16 activating receptors and higher frequency of NK cells expressing the NKG2A inhibitory molecule, were found in TIL. Moreover, Siglec-7, a NK receptor whose reduced frequency is associated with decreased cell function in physiologic (30) and pathologic conditions (31–34), was also reduced.

### Reduced intratumoral NK-mediated ADCC activity in patients with HNSCC

**Figure 4:**
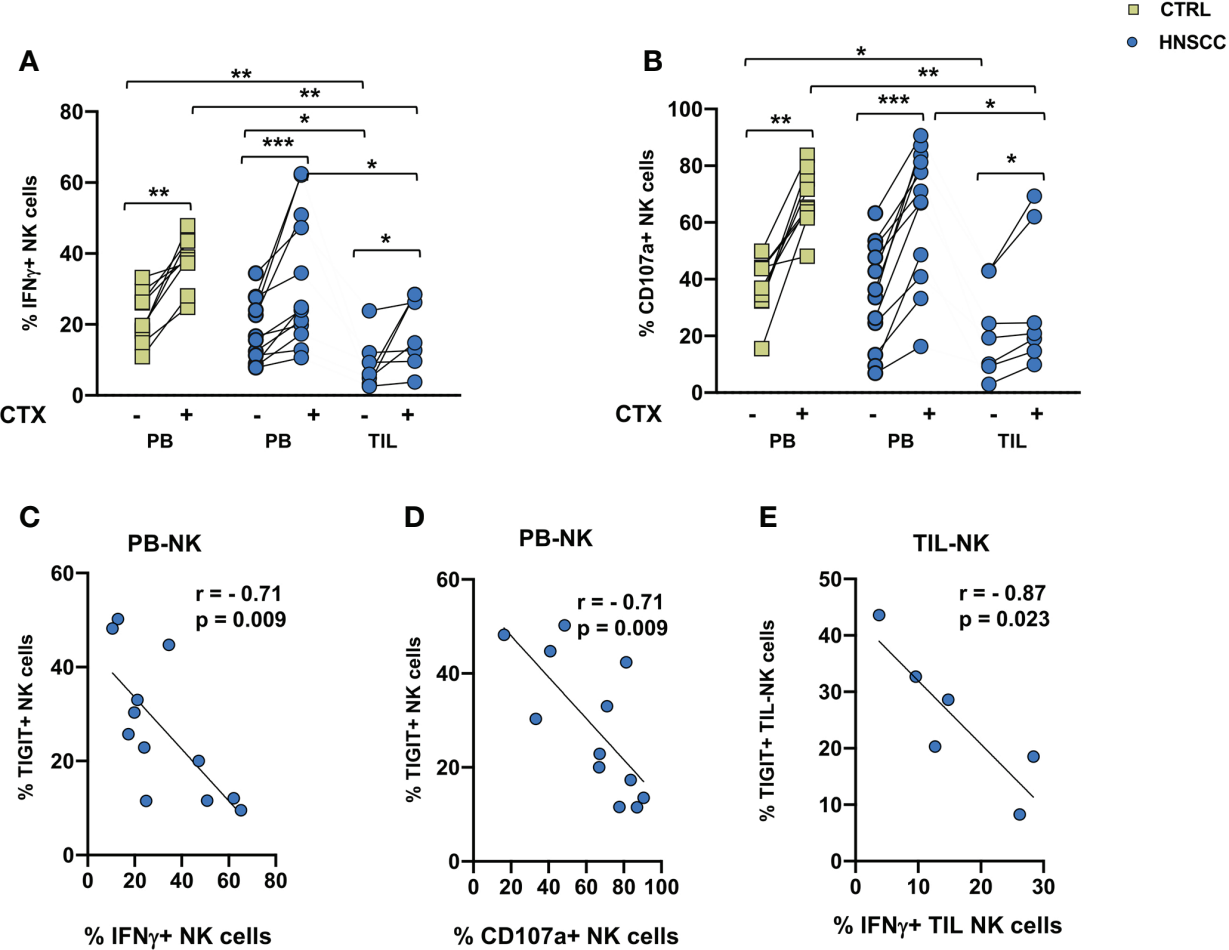
**(A)** IFNγ production and **(B)** degranulation ability by IL12+15+18 pre-activated NK cells following stimulation with SW480 with or without Cetuximab. Paired data were analyzed by the Wilcoxon signed rank test. The non-parametric Kruskal-Wallis followed by Dunn’s multiple test were used to compare different datasets. *p < 0.05, **p < 0.01, ***p < 0.001. **(C)** IFNγ and **(D)** CD107a expression negatively correlated with TIGIT expression on peripheral NK cells. **(E)** Intratumoral IFNγ expression negatively correlate with TIGIT expression on tumor infiltrating NK cells. The Pearson test was used to examine correlations.

A decreased frequency of three strong activating receptors and the increase of NKG2A would support the concept that intratumoral NK cells are dysfunctional. To address this hypothesis, we compared TIL and paired PBMC function, by analyzing NK cell IFNγ production and degranulation activity in the presence of the MHC class I non-expressing K562 cells or with EGFR+ SW480 colon carcinoma target cells in the presence of Cetuximab in an ADCC assay. A cytokine combo including IL12+IL18+IL15 was employed as a stimulus because of their established ability to synergize and induce high levels of IFNγ (49). No changes in CD107a expression or IFNγ secretion was observed in NK cells from controls and patients in the presence of K562 target cells (**Supplementary Figure 2**). As shown if **Figures 4A, B**, spontaneous cytotoxicity or ADCC measured either as IFNγ secretion or as degranulation as a readout was significantly decreased in intratumoral NK cells compared with paired HNSSC peripheral and control NK cells. Cetuximab induced a significant increased activity in controls and patients, both in circulating and intratumor NK cells. IFNγ secretion and CD107a expression of circulating NK cells were inversely correlated to the frequency of TIGIT expressing NK cells (**Figures 4C, D**). IFNγ secretion by TIL-NK cells was inversely correlated to the frequency of TIGIT expressing TIL-NK cels (**Figure 4E**).

### Adaptive NK cell expansion increases ADCC function in HNSCC patients

**Figure 5:**
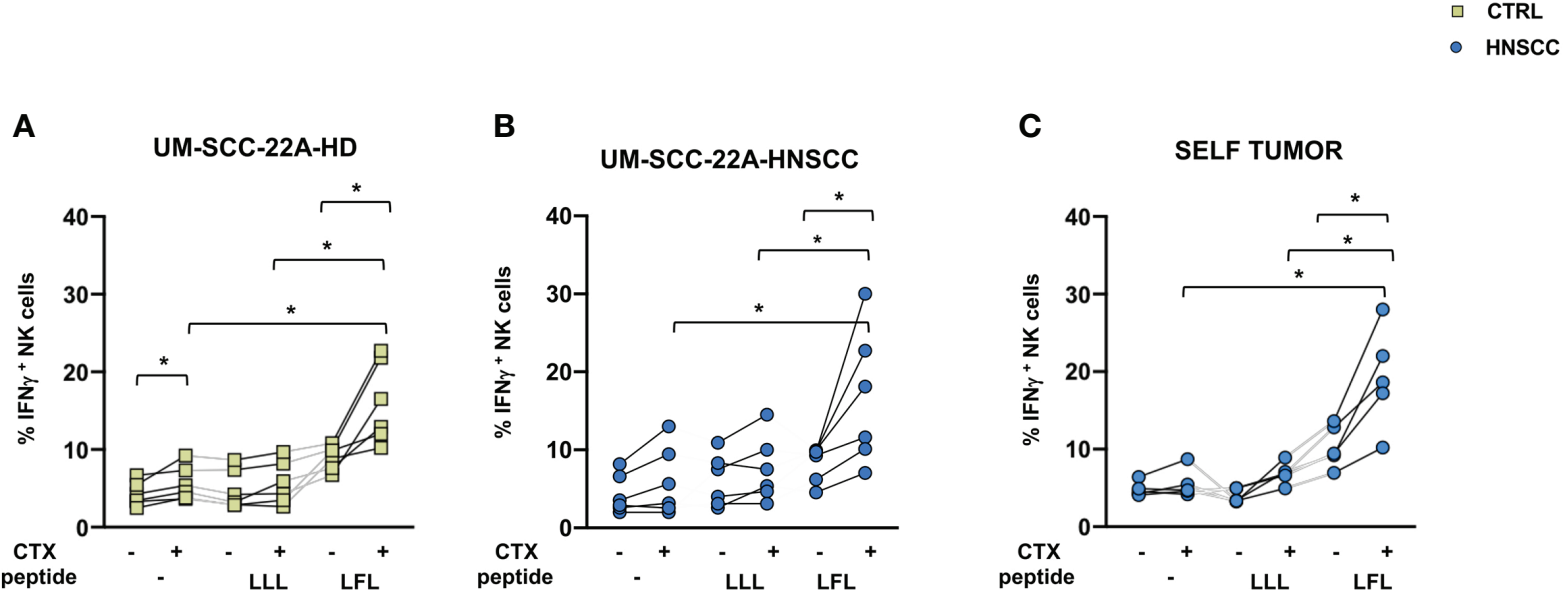
**(A)** IFNγ secretion by control and **(B, C)** HNSCC patient NK cells after 10-day cell culture with 400μm VMAPRTLLL (LLL) or VMAPRTLFL(LFL) UL-40 CMV peptide in HCMV+ individuals. **(A, B)** UM-SCC-22A or **(C)** autologous primary tumor cells were used as targets in an ADCC assay in the presence of Cetuximab or a control antibody. Paired data were analyzed by the Wilcoxon signed rank test. The one-way Friedman followed by Dunn’s multiple comparison tests were used, as appropriate, to compare data within the same group. *p < 0.05.

NK cell effector properties can be exploited in treating certain tumors due to their ability to elicit ADCC. Adaptive NK cells have unique features, being characterized by increased ADCC activity and by resistance to myeloid-derived suppressor cell and Treg-mediated suppression (50, 51). To evaluate the potential of these cells in HNSCC, we expanded this population from PBMC from CMV positive patients by culturing cells in the presence of CMV UL-40 peptide VMAPRTLFL or a control peptide (VMAPRTLLL), and compared the ability of NK cells to secrete IFNγ and CD107a as surrogates of cytotoxicity. We used one commercial EGFR expressing cell lines as target cells, the UM-SCC-2A squamous cell carcinoma of the oral cavity and autologous primary tumor cells. Increased IFNγ secretion by NK cells primed with VMAPRTLFL was observed in controls (**Figure 5A**) and patients (**Figures 5B, C**) compared with unstimulated and control peptide-stimulated cells in the presence of UM-SCC-2A target cells (**Figures 5A, B**) or of primary tumor cells (**Figure 5C**). In contrast, degranulation of CD107a was similar in peptide-primed and unprimed NK cells (**Supplementary Figure 3**). **Supplementary Figure 4** shows EGFR expression in primary tumor cells

### Circulating and tumor infiltrating T cells in HNSCC

**Figure 6:**
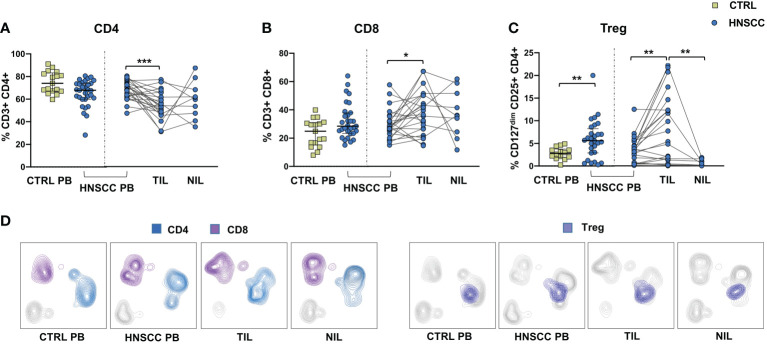
**(A)** Frequencies of CD4+ T cells, **(B)** CD8+ T cells, **(C)** Treg cells in PBMC from healthy donors (CTRL PB) and HNSCC patients (HNSCC-PB) and in matched tumor-infiltrating (TIL) and surrounding non-tumor-infiltrating lymphocytes (NIL). The Mann–Whitney U test was used to compare CTRL and HNSCC subjects. Paired data were analyzed by the Wilcoxon signed rank test. *p < 0.05, **p < 0.01, ***p < 0.001. **(D)** UMAP (Uniform Manifold Approximation and Projection for Dimension Reduction) representation of manually gated CD4, CD8 and Treg cells.

**Figure 7:**
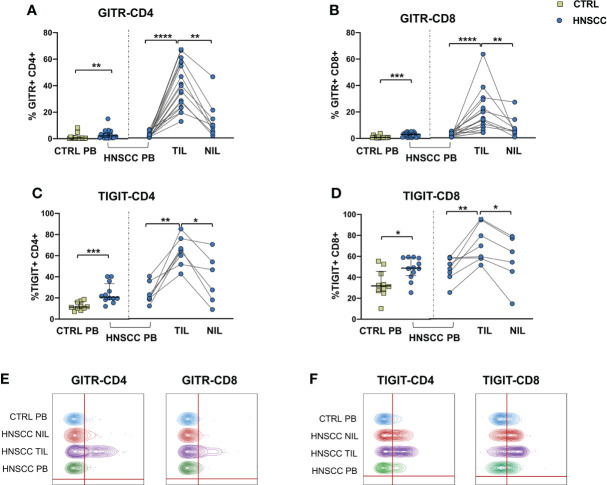
Expression of **(A, B)** the GITR costimulatory molecule and **(C, D)** of the TIGIT checkpoint molecule on CD4+ and CD8+ T cells. The Mann–Whitney U test was used to compare CTRL and HNSCC subjects. Paired data were analyzed by the Wilcoxon signed rank test. *p < 0.05, **p < 0.01, ***p < 0.001, ****p < 0.0001. **(E)** Representative concatenated flow cytometry plots showing the percentage of GITR-expressing CD4+ and CD8+ T cells. **(F)** Representative concatenated flow cytometry plots showing the percentage of TIGIT expression on CD4+ and CD8+ T cells.

**Figure 8:**
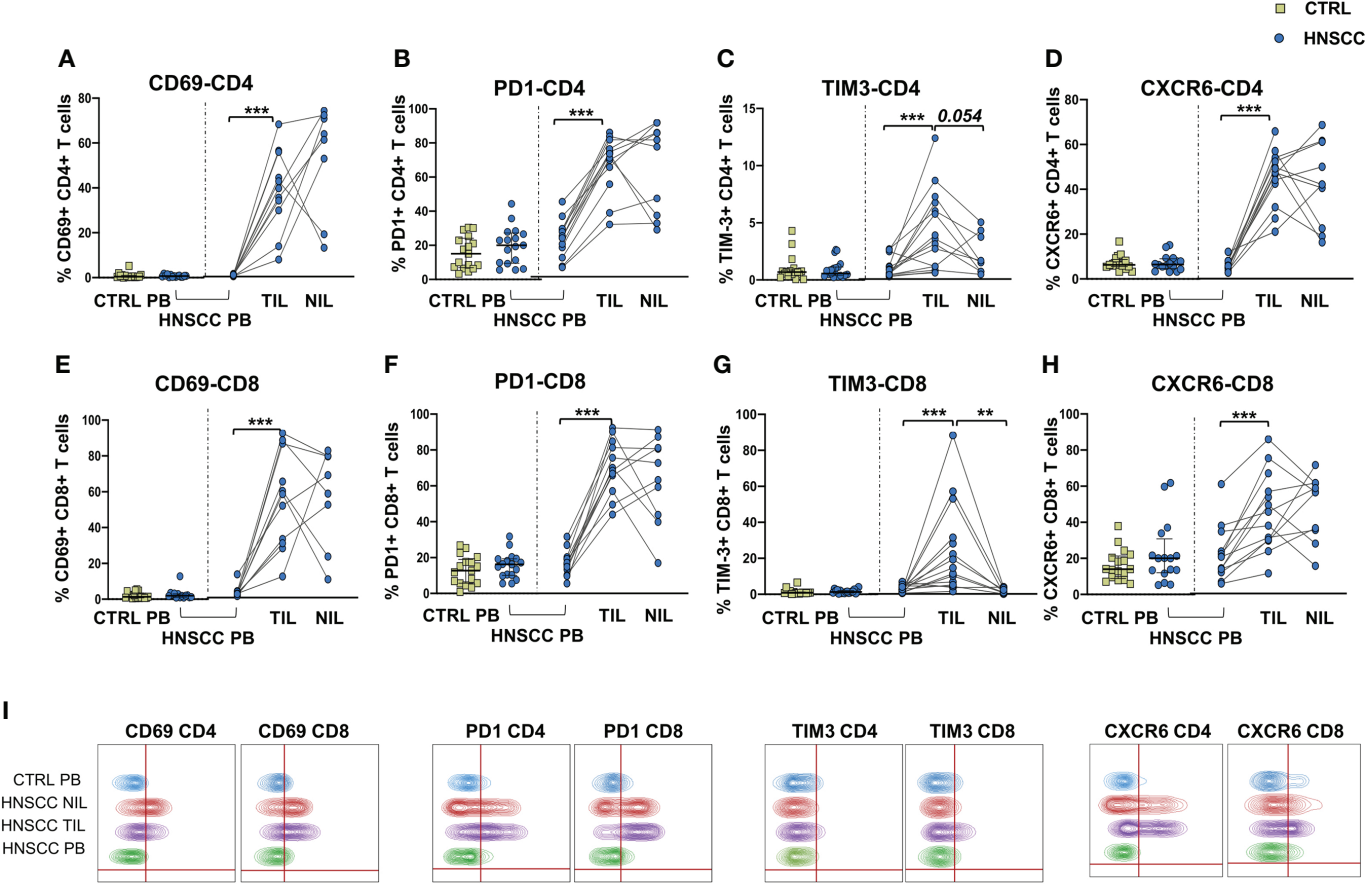
Proportions of **(A)** CD69, **(B)** PD-1-, **(C)** TIM-3-, and **(D)** CXCR6- positive CD4 T cells in PBMC from control subjects (CTRL PB), from HNSCC patients (HNSCC-PB) as well as matched tumor-infiltrating (TIL) and non-tumor-infiltrating lymphocytes (NIL). Frequencies of CD8 T cells expressing **(E)** CD69, **(F)** PD-1-, **(G)** TIM-3-, and **(H)** CXCR6. The Mann–Whitney U test was used to compare CTRL and HNSCC subjects. Paired data were analyzed by the Wilcoxon signed rank test. **p < 0.01, ***p < 0.001. **(I)** Representative concatenated flow cytometry plots showing the percentage of CD4+ and CD8+ T cells expressing the indicated phenotypic markers.

As a corollary of this study, we also compared peripheral and intratumoral T cell phenotype in controls and HNSCC patients. Frequencies of circulating CD4 and CD8 T cells were not different in the two groups, while there was a significant reduction in the proportion of CD4 T helper cells, and an enrichment in the frequency of CD8+ T cells in TIL (**Figures 6A, B, D**). A significantly increased frequency of the regulatory T cell population (Treg) was also observed in the peripheral blood of HNSCC patients compared with controls, and a further significant increment was found in the intratumoral tissue when compared with paired circulating T cells and with non tumoral tissue (**Figure 6C, D**). Moreover, frequencies of circulating CD4 and CD8 T cells expressing the immunostimulatory checkpoint GITR (**Figures 7A, B, E**) or the inhibitory receptor TIGIT (**Figures 7C, D, F**) were significantly increased in HNSCC patients compared with controls. Interestingly, their relative proportions were further increased in TIL compared with PBMC and NIL (**Figure 7**). Moreover, the proportions of intratumoral CD4 and CD8 T cells expressing the CD69 homing marker, as well as PD-1, TIM-3 inhibitory co-stimulatory molecules and CXCR6 were increased compared with the peripheral compartment (**Figure 8**). TIGIT (**Figures 7C, D**) and TIM-3 (**Figures 8C, G, I**) inhibitory receptors were specifically increased in TIL and not in NIL CD4 and CD8 T cells, suggesting that the tumor microenvironment plays a crucial role in inhibiting tumor-specific T-cell responses. Circulating PD-1+,TIM-3+, CD69+ and CXCR6+ CD4 and CD8 T cells frequencies did not differ between the two groups (**Figure 8**).

## Discussion

In this study we have examined the NK and T cell immunophenotype in peripheral blood and in tumor tissue of patients with HNSCC undergoing surgery. Other studies had previously characterized HNSCC circulating immune cells; however most were limited to assessing the distribution of the principal lymphocyte populations (20, 52–54) and only few analyzed NK cell inhibitory and activating receptors (55–57). In agreement with others, we observed reduced frequencies of the immunoregulatory CD56^bright^ NK cell population (58) and increased frequencies of TIM-3+ and NKG2A+ NK cells (56, 59) in the peripheral blood of patients with HNSCC compared with healthy donors. Moreover, the proportion of Siglec-7+ NK cells was significantly reduced in patients. Interestingly, a recent study showed that reduced Siglec-7 expression predicts NK cell dysfunction in hepatocellular carcinoma (60), confirming the role of this receptor in the modulation of NK cell activity, as already observed in physiological and pathological conditions (30–34).

Several recent studies pointed to the relevance of the immune infiltrate in HNSCC, showing the prognostic role of TIL specific markers, including CD56 and CD57 (20, 61, 62), CD3, CD4 and CD8 (63–68). A meta-analysis showed that M2 macrophages and CD57+ natural killer cells were the most promising predictors of survival in oral cancer patients (69).

On this basis, it has been proposed that incorporation of TIL evaluation in the TNM system could improve the prognostic performance, and help physicians in clinical decisions. NK cells are potentially very effective against tumor growth, being able to kill tumor cells when activating signals prevail on inhibitory signals. However, the tumor immunosuppressive microenvironment may inhibit NK cell function by tipping the balance toward inhibition. Our study shows that intratumoral NK cells are characterized by an inhibitory phenotypic pattern, with reduced frequencies of cells expressing the major activating receptors NKG2D, NKp30 and CD16. The importance of NKG2D in tumor control is highlighted by the fact that tumors release NKG2D ligands MICA/B and ULBP1/2/3 by proteolytic cleavage to escape NKG2D-mediated NK cell cytotoxicity (70) and that antibody targeting the site of proteolytic cleavage prevent release of MICA/B and reduce melanoma metastasis in a humanized mouse model (71). This mechanism of immune evasion has also been observed in HNSCC in an interesting study showing that increased levels of soluble NKG2D ligands are present in the plasma of HNSCC patients (72) and that depletion of these ligands from patients’ plasma restores the ability to kill target cells *in vitro* (73). In agreement with others (57, 74) we have observed increased frequencies of NKG2A positive intratumoral NK cells compared with matched PBMC. Different types of tumors overexpress NKG2A and its ligand HLA-E (75, 76), and blocking NKG2A is an effective strategy to enhance antitumor activity by NK and CD8 T cells (77). We observed high level of HLA-E in primary tumor cells obtained from 4 patients with HNSCC (**Supplementary Figure 5**).

Not surprisingly, the functional activity of intratumoral NK cells was reduced, reflecting the prevalent inhibitory phenotype, which was also accompanied by an enrichment in PD-1+ TIGIT+ NK cells

and a reduction in CD16, a receptor capable to bind the antibody Fc fragment, thus enabling NK cells to perform ADCC. Downregulation of CD16 is induced in different tumors and viral infections, through matrix metalloproteinases (MMPs) induced shedding (78, 79), as an escape mechanism to avoid ADCC. Among MMPs, ADAM-17 mediated cleavage has been recognized as responsible of CD16 shedding in breast cancer (78, 80). ADCC process is triggered by Cetuximab, an anti EGFR antibody, which is widely used in cancer immunotherapy, including locally advanced- or recurrent/metastatic HNSCC (4, 81). Cetuximab engagement of CD16 activates NK cells and induces IFNγ secretion (82), an important soluble factor which promotes dendritic cell maturation leading to increased tumor antigen presentation and expansion of EGFR specific T cells (83). Our data show that intratumoral NK cells have reduced ability to secrete IFNγ in the presence of cetuximab after stimulation with a potent combination of cytokines, while peripheral NK cells from patients produced IFNγ similarly to controls. Interestingly, patients with higher frequencies of TIGIT expressing NK cells produced less IFNγ in the peripheral blood and intratumor compartments. TIGIT is a co-inhibitory receptor on the immune cell surface that binds ligands CD155 and CD112 (84). Different strategies are employed to increase NK cell activity against solid tumors in a number of clinical and pre- clinical studies (85). Blocking TIGIT interaction with its ligands reduces NK cell exhaustion *in vitro* and in a murine model (86, 87). Moreover, several anti-TIGIT monoclonal antibodies are under investigation in phase I/II studies (88).

Similarly to NK cells, intratumoral T cells were characterized by an exhausted phenotype, with increased proportions of TIM-3, PD-1 and TIGIT expressing CD4 and CD8 T cells. Moreover, patients’ Treg cells were increased in both the circulating and intratumoral compartments, further supporting the inhibitory role of the TME.

Increased frequencies of GITR+ NK cells were found in TIL and, in agreement with others (89, 90) we also observed a significant enrichment of GITR+ CD4 and CD8 T cells. GITR is a costimulatory receptor which has a dual positive effect supporting the survival and expansion of activated T cells and at the same time deactivating the Treg population within tumors (91). For this reason, GITR represents an ideal targetable immunomodulatory receptor that could provide a powerful tool to diminish the suppressive potential of intratumoral Treg and enhance anti-tumor immunity. Of note, different clinical trials are testing GITR agonist antibodies in combination with immune checkpoint inhibitors in advanced tumors (92–95). On this basis, considering the increased frequencies of NK and T cells expressing GITR, the use of agonistic GITR combined with blocking PD-1 and/or TIGIT antibodies could be beneficial in patients with HNSCC.

Increased proportions of CXCR6 were found in intratumoral NK and T cells, probably caused by ligand-receptor chemotaxis of CXCL16/CXCR6. Interestingly, a recent study in mice showed that CXCR6 is highly expressed in intratumoral CD8+ T cells and that these cells are more immunocompetent and could enhance the effect of anti-PD-1 blockade to delay tumor progression (96). CXCR6 has been recognized as a marker for liver resident NK cells (97) and has been associated to murine CMV virus specific adaptive-memory NK cells (98, 99). Adaptive NK cells have peculiar characteristics that have been exploited in adoptive anticancer therapy, being characterized by increased ADCC activity and by resistance to myeloid-derived suppressor cells and to Treg-mediated suppression (50, 51). Interestingly, a lower leukemia relapse rate and a superior disease-free survival at 1 year has been associated with CMV reactivation and this protective effect strongly correlated with adaptive NK-cell expansion (100). We have observed an increased IFNγ secretion, but not CD107a expression, by NK cells after 12 days expansion of adaptive NK cells in CMV+ patients, confirming that these cells are specialized in IFNγ secretion after Fc binding of therapeutic antibodies and that NK cell expansion by UL40 peptide stimulation combined with IL2 is a feasible way to obtain the expansion of highly functional NK cells in CMV positive subjects.

In conclusion our data show that the HNSCC tumor microenvironment affects NK and T cells, inducing an inhibitory/exhausted phenotype which is reflected in reduced NK cell ability to produce IFNγ after cetuximab engagement. The concomitant increase of costimulatory receptor GITR in intratumoral NK and T cells suggests that agonist engagement of GITR could be an effective treatment in HNSCC patients, particularly if combined with immune checkpoint blocking antibodies anti-PD-1 and anti-TIGIT.

## Data availability statement

The raw data supporting the conclusions of this article will be made available by the authors, without undue reservation.

## Ethics statement

The studies involving human participants were reviewed and approved by Institutional Review Board and Ethical Committee of Fondazione IRCCS Policlinico San Matteo, Pavia, Italiy. Document number 20190063538. The patients/participants provided their written informed consent to participate in this study.

## Author contributions

DM, SV, and MM provided substantial contribution to the conception and design of the study, acquisition, analysis and interpretation of data, and revised the manuscript critically for important intellectual content. SV and MM supervised the team, obtained funding and had leadership responsibility for the research activity planning and execution. BO, SM, and GP contributed to acquisition, analysis, validation and interpretation of data. GT, AL and MB were responsible for data curation and were in charge of patient care. PM performed the histopathologic analysis. All authors critically read, edited and approved the final version of the manuscript.

## Funding

This work was funded by Ricerca Corrente, Ministero della Salute, Nr. 08071419 to SV.

## Acknowledgments

We thank patients and donors included in the study.

## Conflict of interest

The authors declare that the research was conducted in the absence of any commercial or financial relationships that could be construed as a potential conflict of interest

## Publisher’s note

All claims expressed in this article are solely those of the authors and do not necessarily represent those of their affiliated organizations, or those of the publisher, the editors and the reviewers. Any product that may be evaluated in this article, or claim that may be made by its manufacturer, is not guaranteed or endorsed by the publisher.
