# Supplementary figures and images for: Impaired intratumoral natural killer cell function in head and neck carcinoma
Source: Front Immunol. 2022 Oct 20;13:997806. doi: 10.3389/fimmu.2022.997806 (PMC9630640; doi:10.3389/fimmu.2022.997806)

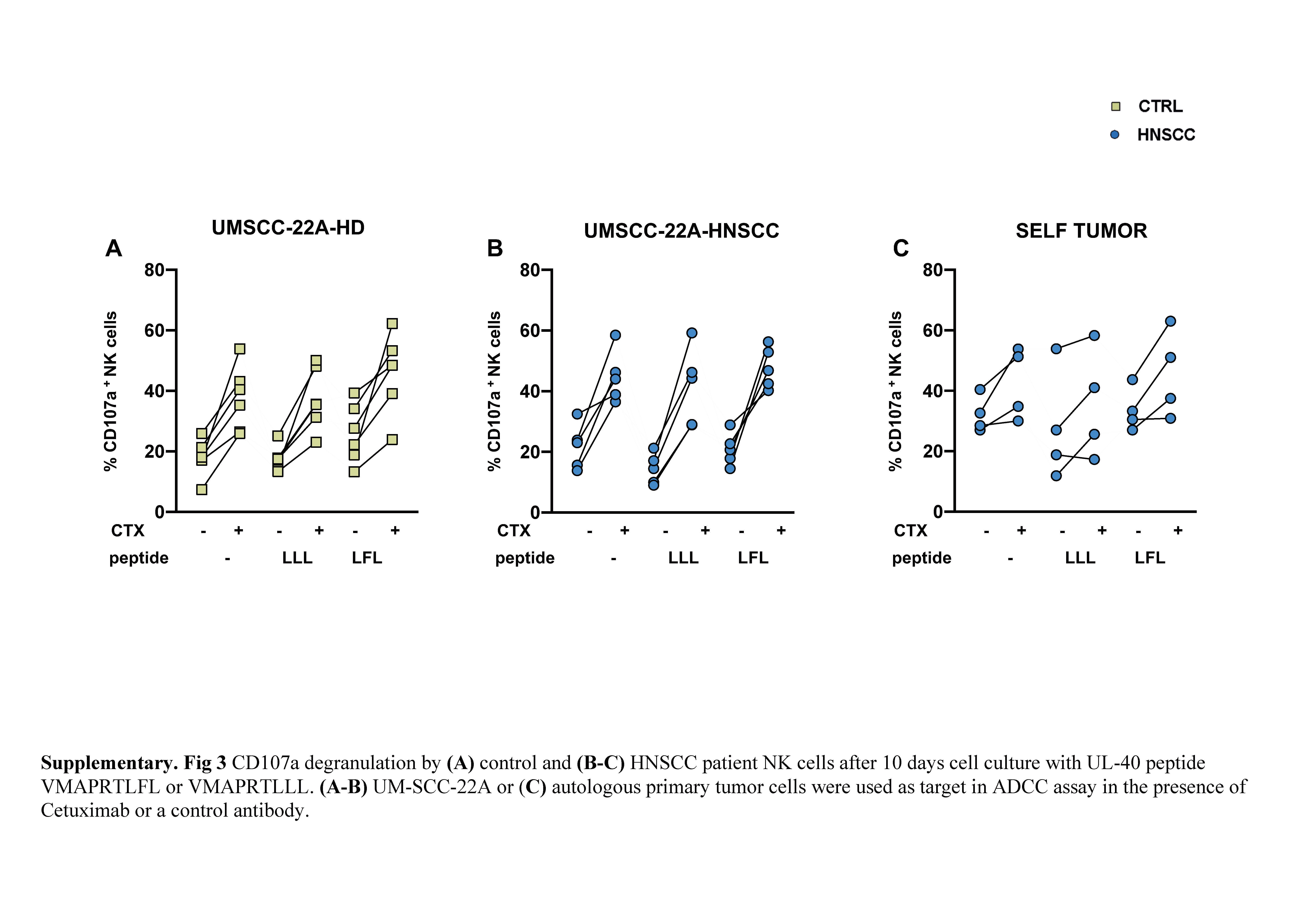

Supplement: Supplementary file 3 [file Image_3.jpeg]

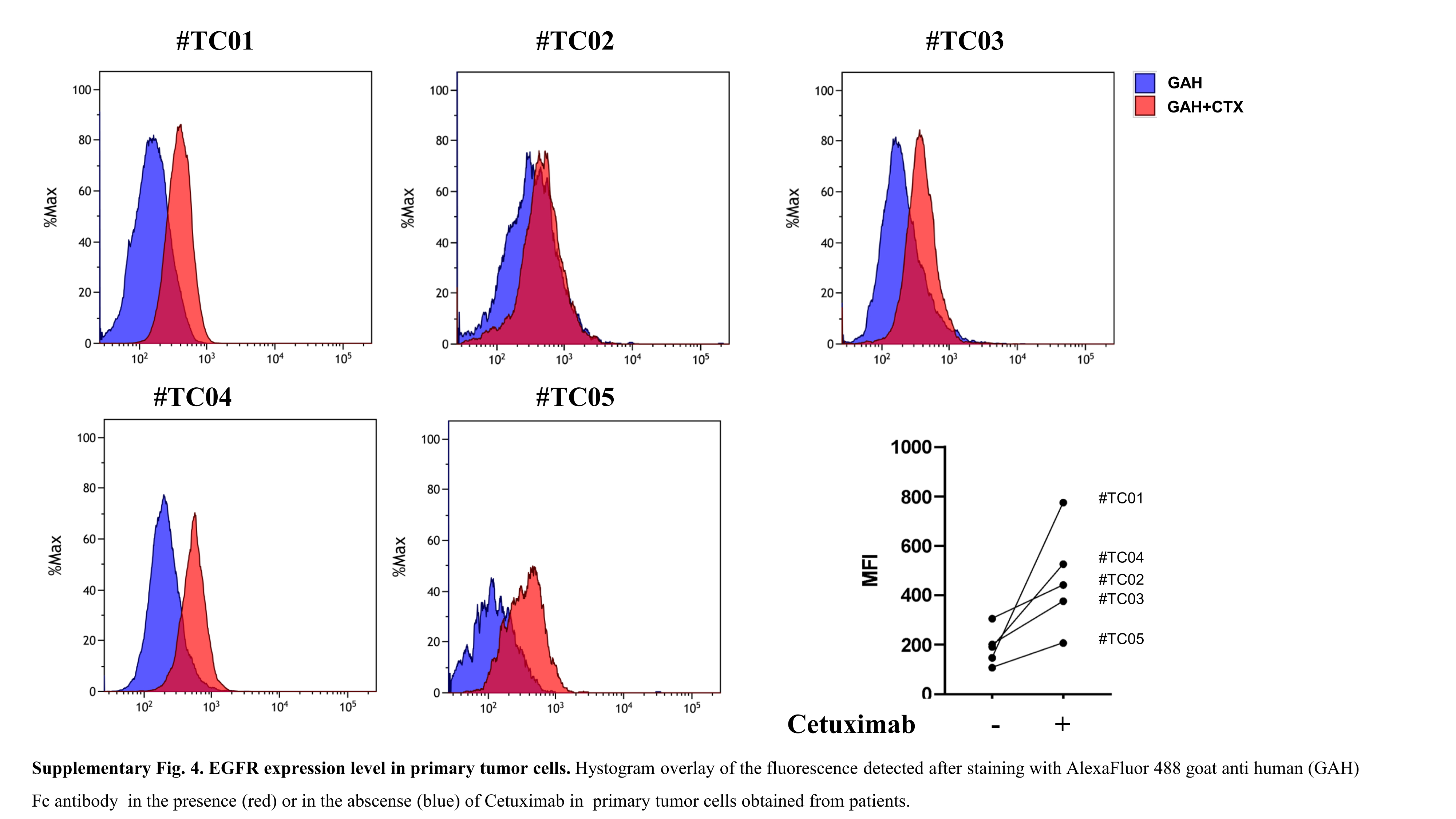

Supplement: Supplementary file 4 [file Image_4.jpeg]

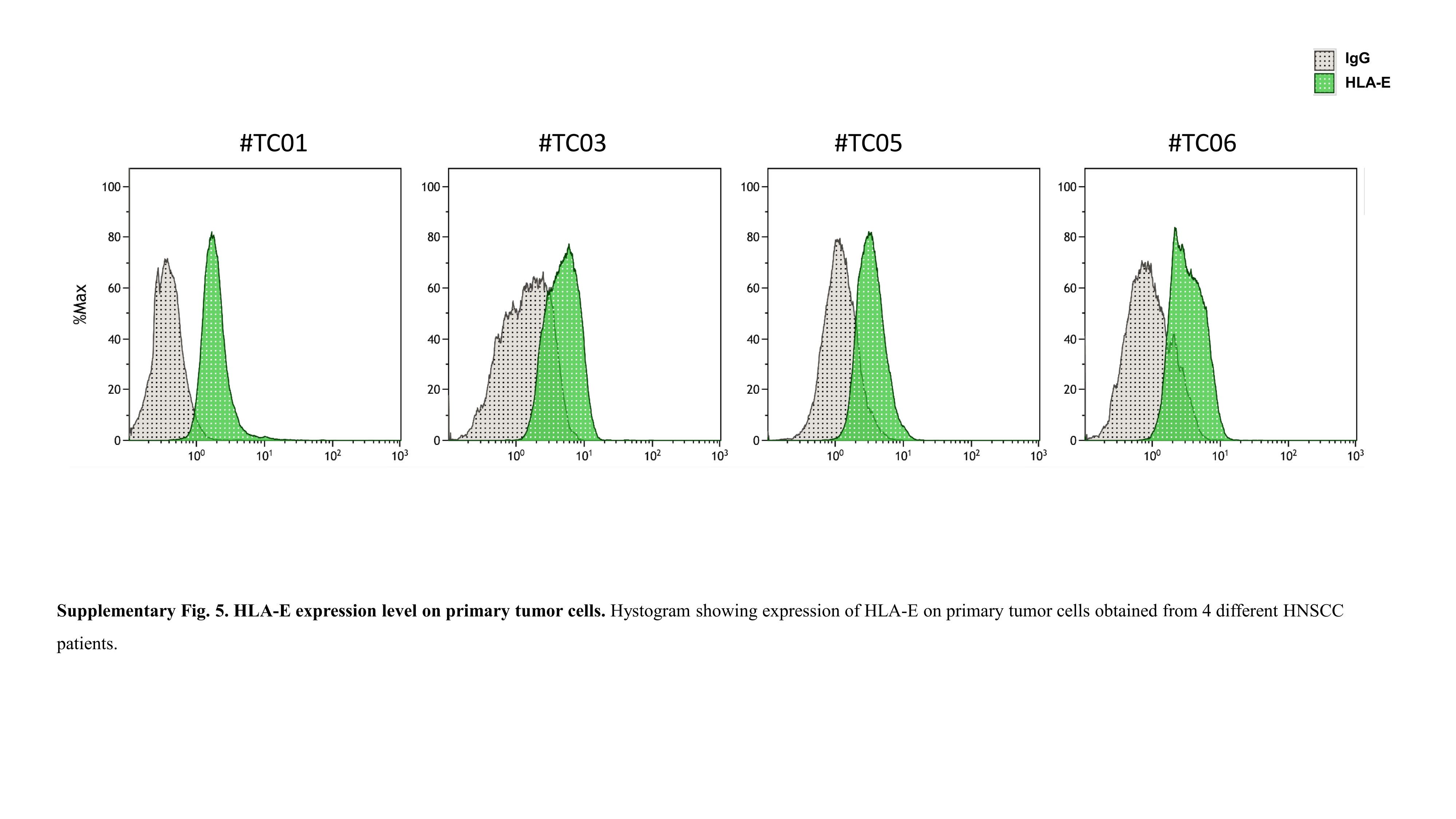

Supplement: Supplementary file 5 [file Image_5.jpeg]
