# Supplementary material for: Impaired intratumoral natural killer cell function in head and neck carcinoma
Source: Front Immunol. 2022 Oct 20;13:997806. doi: 10.3389/fimmu.2022.997806 (PMC9630640; doi:10.3389/fimmu.2022.997806)
Supplement: Supplementary file 6 [file Table_1.docx]

**Supplementary Table 1.**

**Panel A Panel B**

| **Fluorochrome** | **Specificity** | **Clone** | **Vendor** |
| --- | --- | --- | --- |
| **BV421** | **NKG2C** | 134591 | BD |
| **BV605** | **CD3** | UCHT-1 | BD |
| **BV786** | **CD16** | 3G8 | BD |
| **FITC** | **FcεrIγ** | Rabbit polyclonal IgG | Merck Millipore |
| **BB700** | **CD56** | NCAM16.2 | BD |
| **PE** | **Siglec-7** | QA79 | ebioscience |
| **APC** | **CD57** | NK-1 | BD |
| **APC-H7** | **Live-Dead** |  | Thermo Fisher |

| **Fluorochrome** | **Specificity** | **Clone** | **Vendor** |
| --- | --- | --- | --- |
| **BV421** | **NKp46** | 9E2/NKp46 | BD |
| **BV510** | **CD3** | UCHT-1 | BD |
| **BV786** | **NKp30** | p30-15 | BD |
| **FITC** | **FcεrIγ** | Rabbit polyclonal IgG | **Merck Millipore** |
| **BB700** | **CD56** | NCAM16.2 | BD |
| **PE** | **TRAIL** | RIK-2 | BioLegend |
| **PE-CF594** | **NKG2D** | 1D11 | BD |
| **APC** | **NKG2A** | 131411 | R&D System |
| **APC-H7** | **Live-Dead** |  | Thermo Fisher |

**Panel C Panel D**

| **Fluorochrome** | **Specificity** | **Clone** | **Vendor** |
| --- | --- | --- | --- |
| **BV421** | **GITR** | V27-580 | BD |
| **BV510** | **CD3** | UCHT-1 | BD |
| **BV650** | **CD25** | BC96 | BD |
| **BV786** | **CD56** | NCAM16.2 | BD |
| **BB515** | **TIM-3** | 7D3 | BD |
| **PE-CF594** | **CD127** | HIL-7R-M21 | ebioscience |
| **APC** | **CD4** | RPA-T4 | BD |
| **APC-H7** | **Live-Dead** |  | Thermo Fisher |

| **Fluorochrome** | **Specificity** | **Clone** | **Vendor** |
| --- | --- | --- | --- |
| **BV421** | **CXCR6** | 13B1E5 | BD |
| **BV605** | **PD1** | EH12.1 | BD |
| **BV650** | **TIGIT** | 741182 | BD |
| **BV786** | **CD8** | RPA-T8 | BD |
| **FITC** | **FcεrIγ** | UCHT-1 |  |
| **BB700** | **CD56** | NCAM16.2 | BD |
| **PE** | **CD69** | FN50 | BD |
| **APC** | **CD3** | HIT3a | BD |
| **APC-H7** | **Live-Dead** |  | Thermo Fisher |
